# Endothelial function in children with a history of henoch schonlein purpura

**DOI:** 10.1186/s12969-016-0135-z

**Published:** 2017-01-14

**Authors:** Yonatan Butbul Aviel, Lotem Dafna, Giora Pilar, Riva Brik

**Affiliations:** 1 Department of Pediatrics B, Meyer Children’s Hospital, Rambam Medical Center, Haifa, Israel; 2 Pediatric Rheumatology Service, Meyer Children’s Hospital, Rambam Medical Center, Haifa, Israel; 3 Rappaport Faculty of Medicine, Technion-lsrael Institute of Technology, Haifa, Israel; 4 Department of Pediatrics Carmel Medical Center, Haifa, Israel; 5 Meyer Children’s Hospital of Haifa, Rambam Medical Center, Efron Street 1, Bat-Galim, Haifa, 31096 Israel

**Keywords:** Henoch schonlein purpura, Endothelial dysfunction

## Abstract

**Background:**

Although Henoch-Schonlein purpura (HSP) is the most common form of systemic vasculitis in children, the long term effect of HSP on endothelial function is still not clear. The aim of our study was to evaluate the long term effect of HSP on endothelial function in children and adolescents.

**Methods:**

This research was an observational prospective study. The study group comprised of 19 children diagnosed with HSP. The minimum interval between the diagnosis with HSP and endothelial testing was 5 months.

Endothelial function evaluation was assessed by a noninvasive technology named peripheral arterial tonometry, using an EndoPAT™ device. This method measures blood flow in the limb, in response to arterial occlusion, and calculates a Reactive Hyperemic Index (RHI) as an index of endothelial function. RHI values of the study group were compared to those of a known control group.

**Results:**

Nineteen children and adolescents with HSP underwent endothelial function studies. Endothelial function was compared to that of a known control group comprising of 23 healthy children and adolescents. The two groups had similar characteristics, including age, male to female ratio, height, weight and BMI.

Mean RHI was 1.81 in the study group, and 1.87 in the control group (*p* = 0.18). Linear regression of the study group, showed a positive correlation between the time interval from HSP diagnosis to participation in the study, and between the RHI value (r = 0.542, *p* = 0.016). RHI levels were significantly higher in patients who had endothelial function measured more than 6 years since the diagnosis of HSP compared with those patients with less than 6 years follow up (1.98 + 0.74 vs. 1.38 ± 0.43 *P* = 0.037).

**Conclusions:**

These results suggest that HSP causes short term endothelial dysfunction that improves with time.

## Background

Henoch-Schonlein purpura (HSP) is the most common form of systemic vasculitis in children and involves inflammation of the small vessels of the skin, joints, gut, and kidney [1]. Although the precise etiology remains unclear, it has been reported that vascular endothelial function plays a major role in the pathogenesis of HSP.

Recent in vitro studies have demonstrated that markers of endothelial cell damage were elevated in the acute phase of HSP [2, 3]. However, there have been only few clinical studies of assessing the long term damage of vascular endothelium in HSP [4].

The earliest stage of atherosclerosis is based on endothelial dysfunction (ED) [5, 6], since a well functioning endothelium protects the blood vessels from damage by oxidative stress, by release of anti-coagulant, anti-inflammatory, fibrinolytic and vasodilating agents [5]. ED can be basically defined as an impairment of vasodilatation in response to acetylcholine or hyperemia, both of which induce nitric oxide-dependent vasodilatation.

There are several methods to measure endothelial function. In the present study we used Peripheral Arterial Tonometry (PAT), a noninvasive technique that analyzes endothelial function by the measurement of vasodilatation in response to reactive hyperemia [7].

The aim of our study was to assess endothelial function in children with a previous history of Henoch-Schonlein Purpura (HSP) by this non-invasive technique.

## Methods

### Study population

The study includes 19 patients with a previous history of HSP that were previously hospitalized in the pediatric department at Meyer Children’s Hospital, Rambam Medical Center in Israel and were followed in the pediatric rheumatology clinic. Their diagnosis was based on the clinical criteria for HSP [8]. The minimal interval between the diagnosis of HSP and endothelial study was 5 months. Patients with chronic renal disease secondary to HSP or other chronic disease were excluded from the study.

The control group consisted of 23 healthy children with no evidence of structural or functional heart disease, and no medical history of Kawasaki disease or other chronic disorders.

Written informed consent was obtained from all participants and/or their parents. The study was approved by the Rambam Institutional Review Board number 0335-11-RMB.

All subjects were studied in a quiet, temperature-controlled room. All analyses were performed by a single experienced operator.

Anthropometric and physical data for each participant were obtained from the medical charts or were recorded by the investigators during the test (height, weight, blood pressure).

### Assessment of endothelial function

Endothelial dysfunction evaluation was assessed utilizing the EndoPAT device (Itamar Medical Ltd, Caesarea, Israel). This is a noninvasive technology that captures a beat-to-beat plethysmographic recordings of the finger arterial pulse-wave amplitude (PWA) with pneumatic probes. It has been extensively used in our lab both in adults and in children [7, 9–12].

A finger probe is placed on the index finger of each hand, and the peripheral arterial tone (PAT) is recorded from both hands throughout the study. Endothelial function is assessed using the reactive hyperemia technique. Briefly, measurements of the pulse-wave amplitude were obtained during 5 min as baseline (at rest), followed by 5 min of blood flow occlusion in one arm by a cuff that was inflated on the upper arm to suprasystolic pressure (50 mmHg above systolic pressure) and then released to induce reactive (flow-mediated) hyperemia, for 10 min. The other arm remained un-occluded as a reference to correct for potential systemic vasomotor changes.

**Fig. 1:**
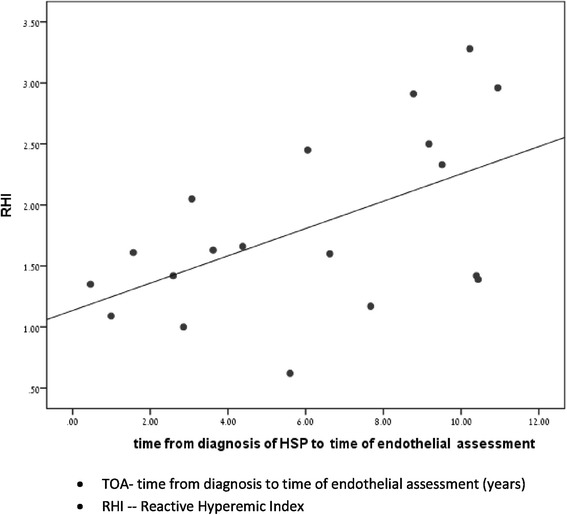
Correlation between TOA* to RHI

Endothelial function is calculated as the ratio between the magnitude of the average post-obstructive PWA (1.5–2.5 min after release of the arterial occlusion) and average 5 min of baseline PWA (pre-occlusion baseline period), corrected to systemic changes (observed at the non-obstructed arm). The threshold for a good EndoPAT result is an RHI of 1.67 and above (Fig 1).

The test was performed in children aged 7 years and above, lying on a comfortable examination table in a cool and quiet room, in the morning or mid- day hours (8:00–14:00).

### Statistical analysis

Statistical analysis included comparisons of PAT values for endothelial function from the study group and the control group, as well as comparisons of clinical data between patients with normal or impaired endothelial function within the study group.

The results are expressed as average ± standard deviation. Pearson correlation was used for the relation between age and RHI levels.

We used the two-tailed *t*-test when normal distribution was assumed. *P* < 0.05 considered as statistically significant.

Linear regression, using standard regression diagnostics was used to look at predictive relationships between continuous variables. All analyses were performed using the SPSS version 18, Inc. Chicago, IL, USA.

## Results

The study group comprised of 19 children and adolescents that were diagnosed with HSP. Endothelial function was compared to that of a known control group of 23 healthy children and adolescents matched controls. Among the control group, there was no differences in RHI values between male and female (1.85 ± 0.35 vs 1.8 ± 0.3 respectively *p* = 0.2) as well as between age and RHI levels in the control group (R-0.29 *P* = 0.18).

The average age in the HSP group was 13.5 ± 3.9 years (range 7–19.7 years) and was similar to the control group 12.8 ± 4.5 years (range 6–23 years) (*p* = 0.4), there were 11/19 male (57%) in the HSP group and 16/23 (69%) male in the control group (*P* = 0.35). BMI was similar in both groups, 22 ± 6.1 in the HSP group and 18.8 ± 3.8 in the control group *p* = 0.13.

Among the children diagnosed with HSP 19 patients (100%) had rash, 17 (89%) had arthritis, 9 (47%) had abdominal pain, and 7 (37%) had hematuria that resolved with time.

Six children (31.6%) were treated with steroids during their hospitalization five of the six patients received steroids for abdominal pain, and for one patient the reason was not specified in the medical record. The average hospitalization were 3.6 ± 2 days (median −3 days).

The average time from the diagnosis of HSP to endothelial function evaluation was 6 ± 3.5 years (median- 6 years). The average RHI value in the patients with HSP was similar to the control group (1.8 ± 0.7 vs 1.87 ± 0.35 p = 0.18). Twelve of 19 (63%) of the children with HSP had abnormal RHI compared with 7/23 (30%) in the control group (*p* = 0.06).

Correlation between RHI levels and different variables were assessed. No correlation was found between RHI and gender (*p* = 0.27), ethnicity (*p* = 0.48), BMI (*p* = 0.84) and clinical features such as abdominal pain (p = 0.62), renal involvement (p = 0.58) and length of stay in the hospital (p = 0.4).

RHI levels among six patients treated with corticosteroids was found to be lower than the group without steroids therapy at the time of the diagnosis of HSP (1.35 ± 0.6 vs 1.9 ± 0.6) (*p* = 0.049).

The average time from diagnosis of HSP to time of endothelial assessment (TOA) was with high correlation to RHI (r = 0.542, *p* = 0.016) (Fig. 1).

**Fig. 2:**
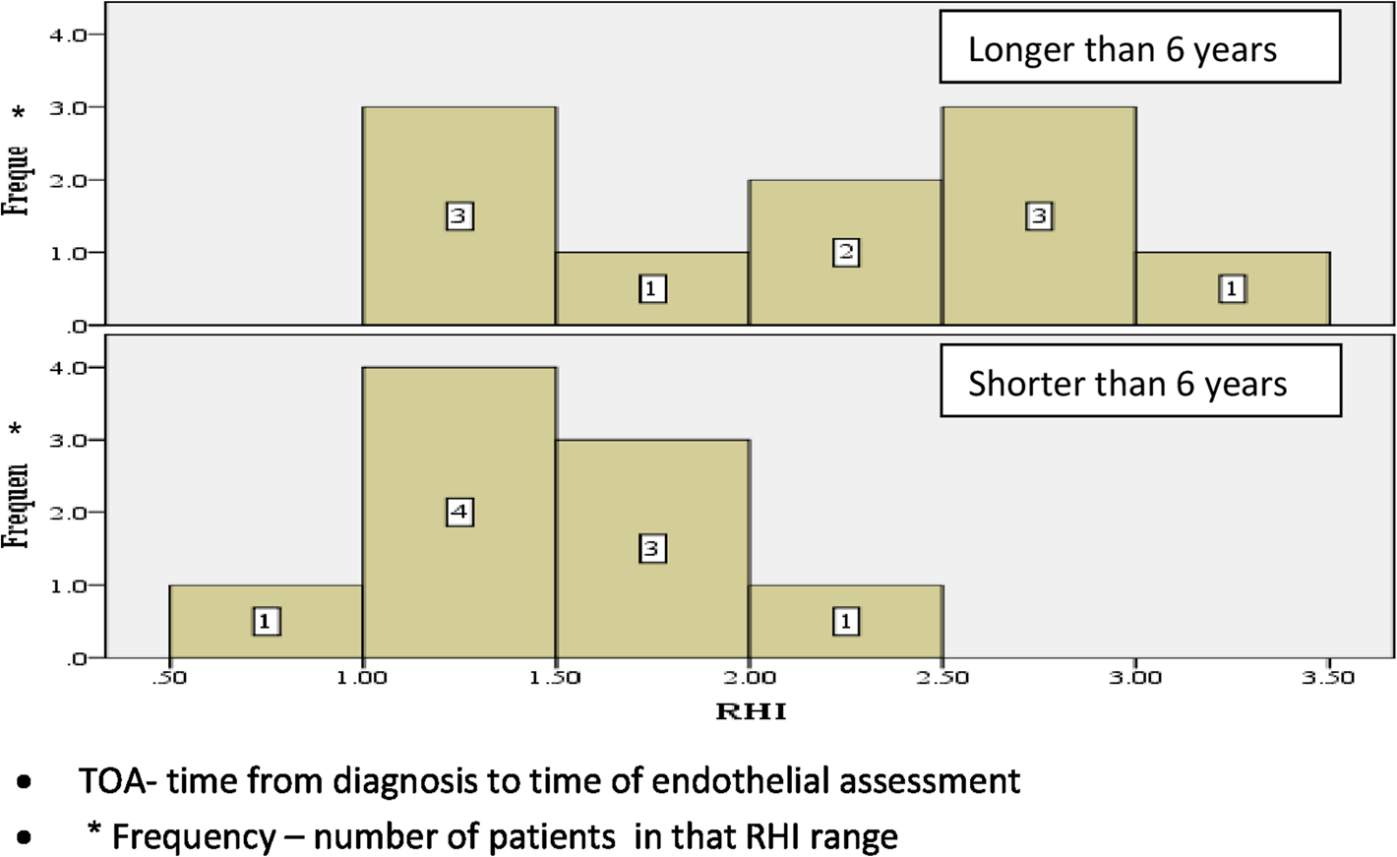
RHI levels among patients with *TOA longer and shorter than 6 years

Patients with TOA longer than 6 years had significantly higher RHI levels compared to those with a period shorter than 6 years (2.2 ± 0.75 vs. 1.38 ± 0.43 *p* = 0.035) (Fig. 2).

In multivariant regression model to predict RHI level by different variables (age, gender, TOA, origin, and steroid therapy), the variable were chosen based on correlation between TOA and gender in the univariable analysis combined with basic demographic data. The final model included gender (female higher) (β = 0.58 *P* = 0.034), and the TOA that had a positive effect on RHI (β = 0.091, *P* = 0.025).

$$ \mathrm{R}\mathrm{H}\mathrm{I} = 2.112 + 0.091*\mathrm{T}\mathrm{O}\mathrm{A} + 0.584*\mathrm{female}. $$

The correlation between TOA and RHI in female was found to be higher than in male although the RHI levels were not different (female r = 0.87 *p* = 0.004 vs male r = 0.37 *p* = 0.26).

When other clinical factors were compared between male and female no differences could be found (Table 1).

**Table 1:** Comparison of different clinical features between genders

	Male	Female	*p*
TOA	6.2 ± 3.7 (6.6)	5.8 ± 3.6 (5.2)	*P* = 0.90
Age at diagnosis of HSP	7.03 ± 2.40 (7.1)	8.08 ± 3.00 (7.2)	*P* = 0.66
Age of endothelial assessment	13.24 ± 3.7 (13.3)	13.9 ± 4.5 (14.2)	*P* = 0.78
Abdominal pain	5 (45.5%)	4 (50%)	*P* = 1.00
Arthritis	9 (82%)	8 (100%)	*P* = 0.49
Renal involvement	6 (54.5%)	1 (12.5%)	*P* = 0.15
Steroids treatment	3 (30%)	3 (37.5%)	*P* = 1.00

## Discussion

The vascular endothelium is not only a semi-permeable barrier between the blood and the interstitium but also a homeostatic organ with physiological, biological, and endocrine functions. In adults, several studies have investigated the peripheral circulation using the forearm hyperemic response in patient with risk factors for atherosclerosis [13–16]. These studies have demonstrated that peripheral vascular endothelial function plays a crucial role in preventing the progression to coronary arteriosclerosis. However, in children, there have been only a few clinical reports of endothelial dysfunction following vasculitis such as Kawasaki disease as well as following other inflammatory disease such as SLE and JIA [17, 18].

In the present study, we demonstrated the attenuated flow-mediated responses (endothelium-dependent vasorelaxation) in the recovery and long term phase of children with HSP.

HSP is an inflammatory disease that affect the small vessels, but unlike other inflammatory diseases such as SLE and RA, the disease is usually of brief duration and in most of the cases leaves no damage in the target organs [1].

A previous study demonstrated endothelial damage in the acute and subacute phase of patients with HSP [4]. Our study is the first to evaluate the long term effect of HSP on endothelial function.

There are few methods to evaluate endothelial function. Quantitive coronary angiography is the most accurate method. In this method during angiography the coronary response to acetylcholine injection is evaluated [19]. However this is an invasive method and not suitable for children. A second alternative, brachial artery ultrasound scanning, is a noninvasive method using Doppler to measure the brachial artery diameter before and after manometric cuff was inflated to supra-systolic pressure [6, 20].

In this study, as in previous studies, we used the Peripheral Arterial Tonography –PAT, a non invasive technology that captures a beat-to-beat plethysmographic recordings of the finger arterial pulse-wave amplitude (PWA) with pneumatic probes before and after blood flow occlusion. In adults it has been demonstrated that endothelial function as measured by the PAT is highly correlated with the brachial artery ultrasound measurements [21].

This methods was previously used is several studies among children [21–24] and based on those studies normal RHI levels was set to be ≥1.67.

RHI levels in patients with a history of HSP were similar to the control group. This finding fits with the known history of patients with HSP who usually develop no long-term damage in the affected organs. We demonstrate that more than half of patients with HSP had abnormal endothelial dysfunction compared to only 30% in the control group although this differences didn’t reach significance (*p* = 0.06). Our results suggest that endothelial damage may take a long time to recover possibly years. The only clinical feature that correlated with decreased RHI was corticosteroid therapy, which may suggest that patients with severe HSP may have greater endothelial damage. Several in vitro studies [25–27] demonstrated that circulating serum growth factor, adhesion molecules, or other markers of endothelial cell damage were elevated in the acute phase of HSP, especially in severe forms developing IgA nephritis. These vascular abnormalities reduce the availability of nitric oxide, which induces structural and mechanical changes, and may offer important insights into the development and progression of vasculitis initiated end-organ damage. The present study did not include patients with renal impairment, since we wanted to rule out other factors that may affect the endothelial function. Further study is needed to clarify differences between patients with and without renal impairment.

Considering that endothelial dysfunction can be the initial feature of atherosclerosis, and considering that atherosclerosis is a progressive process that can be delayed, halted or even be induced to regress [28], other studies on the effect of HSP on atherosclerosis may be needed.

The main limitation of our study is the small number of patients studied. This may explain why we couldn’t demonstrate RHI differences between the patients with HSP and the control group.

Another limitation is the population studied as we evaluate only patients that were hospitalized due to HSP. Those are usually patients with a more severe HSP course in which there may have been greater damage to their endothelium.

The last limitation could be regarding the RHI levels at different ages. This can effect RHI data when comparing patients with more than 6 years since the diagnosis of HSP compared with those with less than 6 years. There is limited data on the influence of age on RHI and in the control group and so we couldn’t defined any differences in RHI levels related to age.

## Conclusion

Our study was the first to demonstrate that HSP may have long term effect on endothelial function. These changes improve gradually and are not permanent. Further larger studies may be needed to assess the effect of HSP on early development of atherosclerosis.

## Abbreviations

HSP: Henoch schonlein purpura
PAT: Peripheral arterial tone
RHI: Reactive hyperemic index
